# A label-free and enzyme-free platform with a visible output for constructing versatile logic gates using caged G-quadruplex as the signal transducer†
†Electronic supplementary information (ESI) available: Experimental details and supplementary figures. See DOI: 10.1039/c7sc04007e


**DOI:** 10.1039/c7sc04007e

**Published:** 2017-10-20

**Authors:** Junhua Chen, Jiafeng Pan, Shu Chen

**Affiliations:** a Guangdong Key Laboratory of Integrated Agro-environmental Pollution Control and Management , Guangdong Institute of Eco-environmental Science & Technology , Guangzhou 510650 , China . Email: 222chenjunhua@163.com

## Abstract

A complete set of binary basic logic gates (OR, AND, NOR, NAND, INHIBT, IMPLICATION, XOR and XNOR) is realized on a label-free and enzyme-free sensing platform using caged G-quadruplex as the signal transducer. In the presence of an appropriate input, the temporarily blocked G-rich sequence in the hairpin DNA is released through cleavage by the synergetically-stabilized Mg^2+^-dependent DNAzyme which can be made to function *via* the input-guided cooperative conjunction of the DNAzyme subunits. In the presence of hemin, the unblocked G-quadruplex DNAzyme catalyzes the oxidation of 3,3′,5,5′-tetramethylbenzidine (TMB) by H_2_O_2_ to generate a colored readout signal which can be readily distinguished by the naked eye. This strategy is quite versatile and straightforward for logic operations. Two combinatorial gates (XOR + AND and XOR + NOR) are also successfully fabricated to demonstrate the modularity and scalability of the computing elements. The distinctive advantage of this logic system is that molecular events in aqueous solution could be translated into a color change which can be directly observed by the naked eye without resorting to any analytical instrumentation. Moreover, this work reveals a new route for the design of molecular logic gates that can be executed without any labeling and immobilization procedure or separation and washing step, which holds great promise for intelligent point-of-care diagnostics and in-field applications.

## Abstract 2

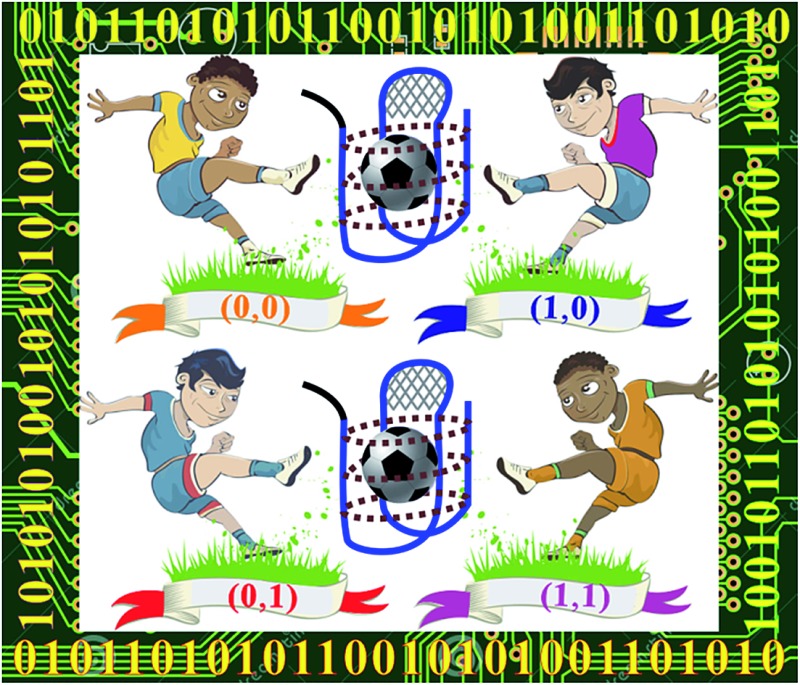
A complete set of elementary logic gates and two multilevel circuits have been constructed on a label-free and enzyme-free biocomputing platform using caged G-quadruplex as the signal transducer.

## Introduction

Molecular logic gates that are capable of performing computational operations could be of use in chemical/biological sensing, disease diagnostics and intelligent imaging, and thus have attracted significant research interest.^1^–^7^ Up to now, various basic logic gates, advanced circuits and even neural networks have been successfully realized, which prove the potential of molecular logic gates in biocomputing science.^8^–^14^ However, the requirement for complex labeling or modifying procedures,^15^,^16^ perishable protein enzymes^17^,^18^ and instrument-dependent readout^19^,^20^ impedes the development of molecular computing and its applications in point-of-care (POC) detection and on-site monitoring. Thus, the establishment of a label-free and enzyme-free sensing platform for constructing versatile logic gates without resorting to any analytical instrumentation is attractive and urgently needed.

Peroxidase-mimicking G-quadruplex DNAzyme is an ideal signal transducer for the construction of DNA logic gates and functional DNA devices.^21^–^25^ Upon incubation with hemin, the formed G-quadruplex/hemin hybrid is able to catalyze the oxidation of 3,3′,5,5′-tetramethylbenzidine (TMB) by H_2_O_2_ and to induce the change in color from colorless to blue.^26^,^27^ In comparison with the instrumentation-dependent fluorescent and electrochemical signals for indirect outputs,^28^,^29^ G-quadruplex provides a direct and visible readout which can be recognized readily by the naked eye.^30^,^31^ The catalytic G-quadruplex structure originates from unblocked G-rich single-stranded DNA, the formation of which can be controlled through hybridization and displacement reactions of caged G-rich sequences.^32^,^33^ In recent years, some elegant sensing platforms have been successfully developed using G-quadruplex as the signal reporter.^34^–^38^ However, there is no trial for the fabrication of a complete set of logic gates, particularly for integrated circuits using caged G-quadruplex as the signal transducer to give out visible results. In this study, we constructed a series of logic gates on a label-free and enzyme-free sensing platform using caged G-quadruplex as the signal transducer. The logic events in the system can be easily transformed into color changes which can be monitored by the naked eye. Our proposed logic strategy herein is simple in operation without any labeling and immobilization procedure or separation and washing step, requiring only the mixing of several solutions at room temperature to obtain intuitive and visible outputs and so it holds great promise for POC detection and on-site monitoring.

## Results and discussion

**Fig. 1:**
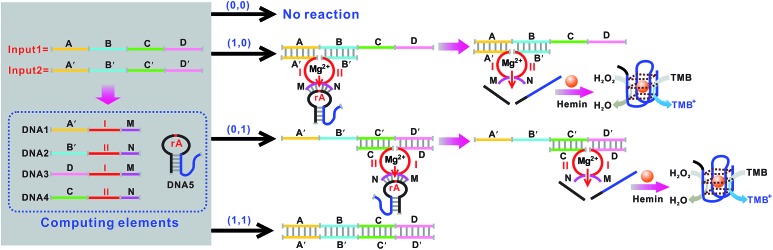
Schematic representation of the XOR logic gate that consists of the DNAzyme subunits (DNA1–DNA4), the substrate (hairpin DNA5, the caged G-quadruplex sequence in the stem structure of the hairpin is indicated in blue) and the input DNA. Throughout the paper the domains X and X′ in the DNA strands represent complementary base pair regions. Domains I and II are the catalytic core components of the Mg^2+^-dependent DNAzyme subunits.

**Fig. 2:**
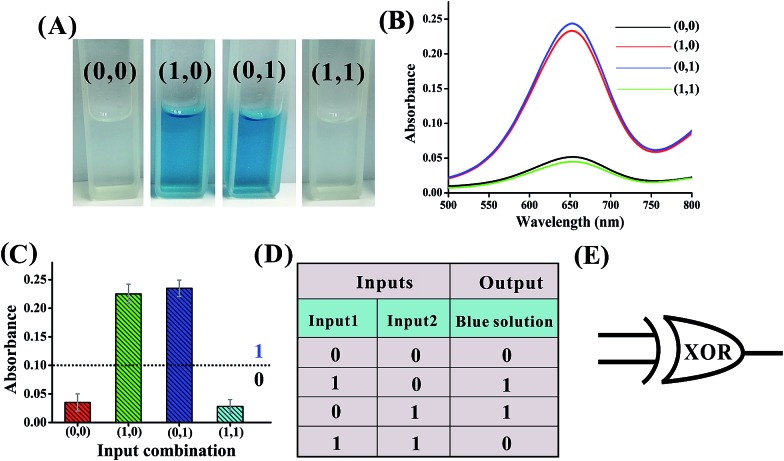
(A) Photographs of the XOR gate with different combinations of inputs. (B) The absorption spectra of this logic gate. (C) Column diagram of the corresponding absorption intensities at *λ* = 650 nm. The black dashed line shows the threshold value of 0.1. (D) Truth table of the XOR gate. (E) Electronic equivalent circuitry.

Mg^2+^-dependent DNAzyme^39^–^41^ subunits (domains I and II) are employed as the computing elements to construct a universal set of logic gates. The separated subunit cannot assemble to form an active DNAzyme. In the presence of appropriate input DNA, the subunits may assemble into active DNAzyme structures *via* input-guided cooperative hybridization processes. The resulting DNAzyme cleaves a ribonucleobase (rA)-containing DNA substrate in the presence of Mg^2+^ to generate output signals. Fig. 1 depicts the construction of the XOR logic gate. The system consists of the DNAzyme subunits DNA1/DNA2 and DNA3/DNA4, the substrate DNA5 and the input DNA. In the absence of an input (0, 0), the subunits cannot spontaneously form any active DNAzyme structure. In the presence of input1 (1, 0) or input2 (0, 1), two different DNAzymes are formed, leading to the cleavage of the hairpin DNA substrate at the ribonucleobase site. The caged G-quadruplex horseradish peroxidase-mimicking DNAzyme sequence (the blue segment in DNA5) in the stem structure of the hairpin is then released and activated. Upon incubation with hemin, the G-quadruplex DNAzyme catalyzes the oxidation of TMB by H_2_O_2_ to generate a colored readout signal which can be readily distinguished by the naked eye. In the presence of both inputs (1, 1), a duplex between inptut1 and input2 will be formed since they are complementary to each other. In this case, the formation of a catalytically active DNAzyme is prohibited. Fig. 2A shows typical photographs of the XOR gate. Fig. 2B depicts the absorption spectra from 500 to 800 nm. Fig. 2C shows the corresponding absorption intensity at *λ* = 650 nm. An absorption intensity of 0.1 is defined as the threshold value for all the logic gates to judge the positive and negative output signals. When the absorption value at *λ* = 650 nm is lower than the threshold value of 0.1, the output of the computing system is considered to be “0” (false output signal). If the absorption intensity is higher than 0.1, the output reads “1” (true output signal).

The solution of the system turned blue (output = 1) only upon activation by input1 or input2 alone, which characterizes an XOR gate. The truth table and circuitry are given in Fig. 2D and E, respectively.

**Fig. 3:**
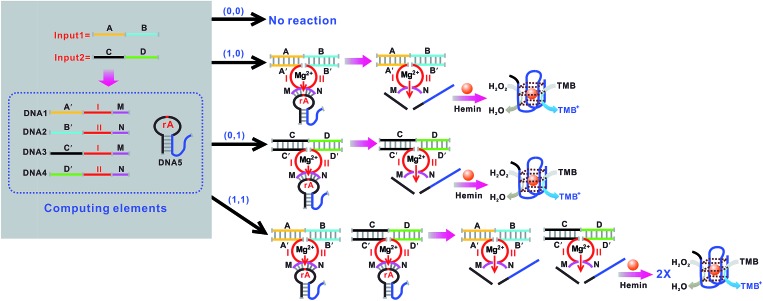
Schematic representation of the OR logic gate that consists of the DNAzyme subunits (DNA1–DNA4), the substrate (hairpin DNA5, the caged G-quadruplex sequence in the stem structure of the hairpin is indicated in blue) and the input DNA.

**Fig. 4:**
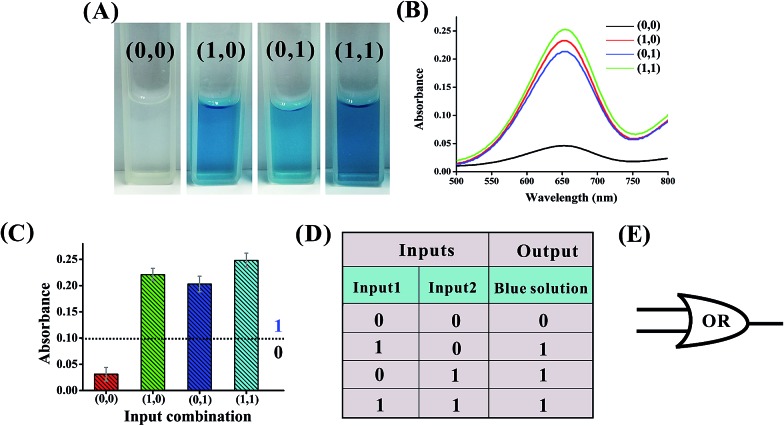
(A) Photographs of the OR gate with different combinations of inputs. (B) The absorption spectra of this logic gate. (C) Column diagram of the corresponding absorption intensities at *λ* = 650 nm. The black dashed line shows the threshold value of 0.1. (D) Truth table of the OR gate. (E) Electronic equivalent circuitry.

An OR logic gate is fabricated by using four DNAzyme subunits (DNA1–DNA4) and the hairpin substrate (DNA5) as the computing elements. As depicted in Fig. 3, input1 is complementary to the recognition arms of the DNAzyme subunits DNA1 and DNA2, and input2 is complementary to the recognition arms of the DNAzyme subunits DNA3 and DNA4. Hence, the addition of either input1 or input2, or both inputs, causes the formation of one or two catalytic DNAzymes which are cooperatively stabilized by the input/substrate components, leading to the cleavage of hairpin DNA5 and to the generation of the colorimetric signals. Fig. 4A shows typical photographs of the OR gate. Fig. 4B depicts the absorption spectra from 500 to 800 nm. Fig. 4C shows the corresponding absorption intensity at *λ* = 650 nm. The OR logic gate is represented by the situation where the output is 1 if either or both inputs are 1. The truth table and circuitry are given in Fig. 4D and E, respectively.

**Fig. 5:**
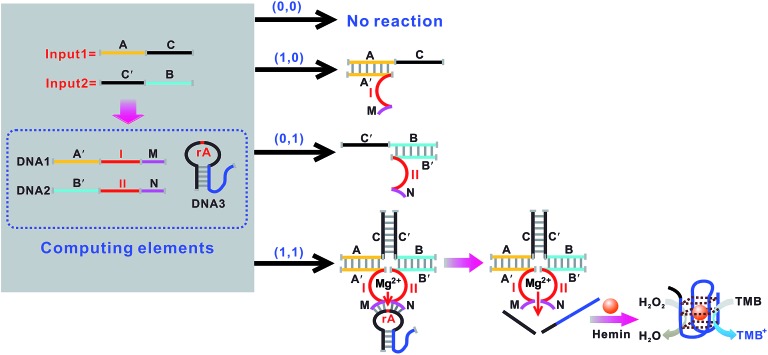
Schematic representation of the AND logic gate that consists of the DNAzyme subunits (DNA1 and DNA2), the substrate (hairpin DNA3, the caged G-quadruplex sequence in the stem structure of the hairpin is indicated in blue) and the input DNA.

**Fig. 6:**
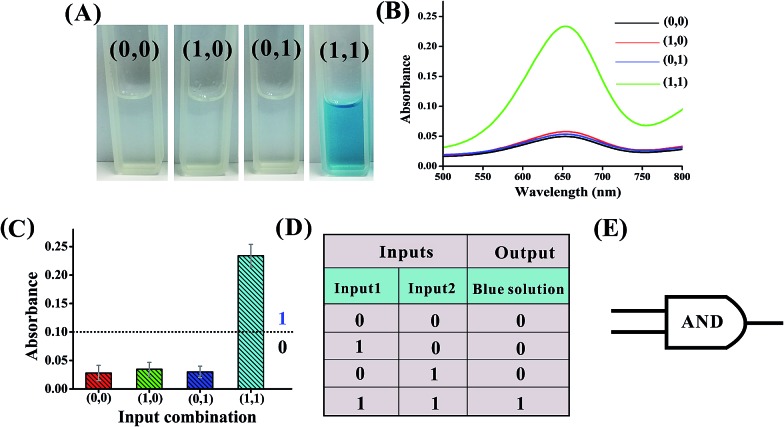
(A) Photographs of the AND gate with different combinations of inputs. (B) The absorption spectra of this logic gate. (C) Column diagram of the corresponding absorption intensities at *λ* = 650 nm. The black dashed line shows the threshold value of 0.1. (D) Truth table of the AND gate. (E) Electronic equivalent circuitry.

Fig. 5 shows the construction of the AND logic gate. This system consists of two DNAzyme subunits (DNA1 and DNA2), the substrate DNA3 and the input DNA. Input1 and input2 are both partially complementary to the two subunits, thus when either input1 or input2 is added to the AND gate individually, these inputs alone cannot assemble the subunits into an active DNAzyme. Such partial hybridization leads to a colorless solution and the output reads 0. In the presence of both inputs (1, 1), cross hybridization between the segments C and C′ of the inputs allows the cooperative binding of the two subunits that results in a synergistically-stabilized active DNAzyme. Fig. 6A shows typical photographs of the AND gate. Fig. 6B depicts the absorption spectra from 500 to 800 nm. Fig. 6C shows the corresponding absorption intensity at *λ* = 650 nm. The AND logic gate is represented by the situation where the output is 1 only if both inputs are 1. The truth table and circuitry are given in Fig. 6D and E, respectively. To investigate the effects of pH and ion strength on the response of the logic system, the (1, 1) state of the AND logic gate was tested under different pH values and ion strengths (Fig. S1, ESI†). Taking the AND logic gate as an example, the formation of the G-quadruplex structure in the logic system was verified by circular dichroism (CD) spectroscopy (Fig. S2, ESI†).

**Fig. 7:**
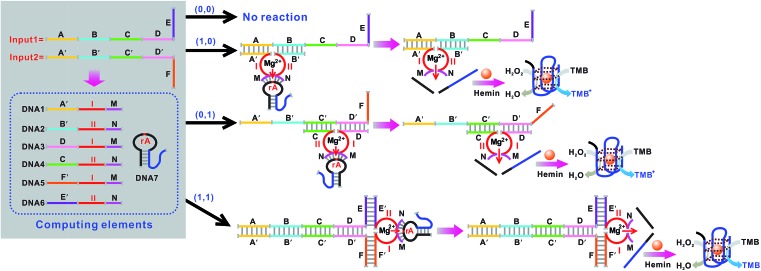
Schematic representation of the XOR + AND logic gate that consists of the DNAzyme subunits (DNA1–DNA6), the substrate (hairpin DNA7, the caged G-quadruplex sequence in the stem structure of the hairpin is indicated in blue) and the input DNA.

**Fig. 8:**
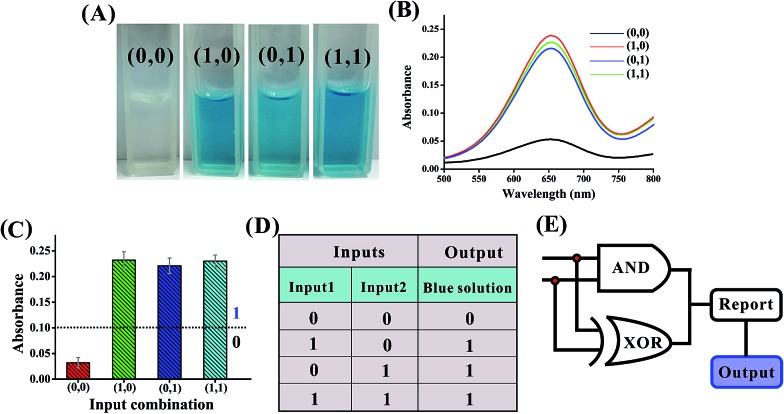
(A) Photographs of the XOR + AND gate with different combinations of inputs. (B) The absorption spectra of this logic gate. (C) Column diagram of the corresponding absorption intensities at *λ* = 650 nm. The black dashed line shows the threshold value of 0.1. (D) Truth table of the XOR + AND gate. (E) Electronic equivalent circuitry.

The basic logic gates could actually be correlated for demonstrating higher-order functions like combinatorial circuits (the construction of the XNOR, NAND, NOR, INHIBIT and IMPLICATION logic gates is depicted in Fig. S3–S12 in the ESI†). Fig. 7 illustrates the operation of the combined XOR and AND logic gate that enforces an overall OR logic behavior. In the presence of either input1 or input2, two active DNAzymes are formed through the input-guided cooperative hybridization of the respective DNAzyme subunits (DNA1, NDA2, DNA3 and DNA4). This leads to the cleavage of the substrate and yields a true output. On triggering the system with both inputs, the four DNAzyme subunits (DNA1–DNA4) cannot assemble into an active DNAzyme structure due to the preferred inter-input hybridization. However, the hybridization between the two inputs can draw the domains E and F together and induce the formation of another synergistically-stabilized active DNAzyme from the DNA5/DNA6 subunits. Thus, an integrated circuit established from the XOR and AND gates could perform in parallel in a single test tube using the same set of inputs. Fig. 8A shows typical photographs of the XOR + AND gate. Fig. 8B depicts the absorption spectra from 500 to 800 nm. Fig. 8C shows the corresponding absorption intensity at *λ* = 650 nm. The combinatorial circuit has an output of 1 if either or both input signals are 1 and so it behaves like the OR gate in operation. The truth table and circuitry are given in Fig. 8D and E, respectively.

**Fig. 9:**
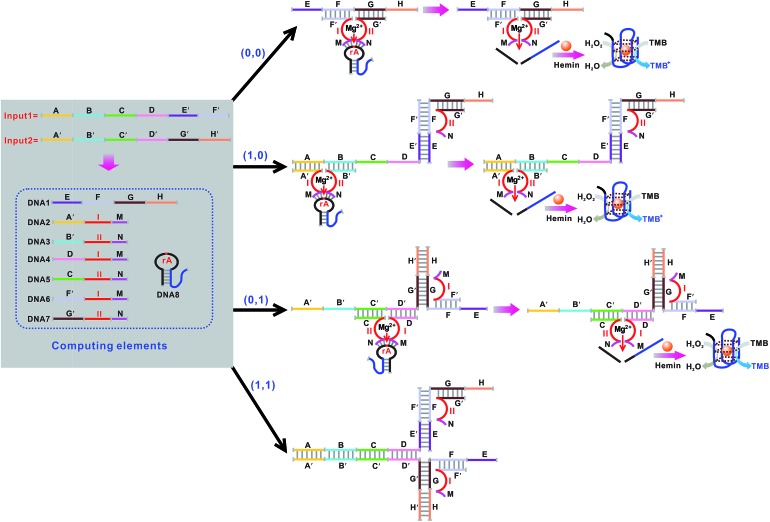
Schematic representation of the XOR + NOR logic gate that consists of the DNA template (DNA1), the DNAzyme subunits (DNA2–DNA7), the substrate (hairpin DNA8, the caged G-quadruplex sequence in the stem structure of the hairpin is indicated in blue) and the input DNA.

**Fig. 10:**
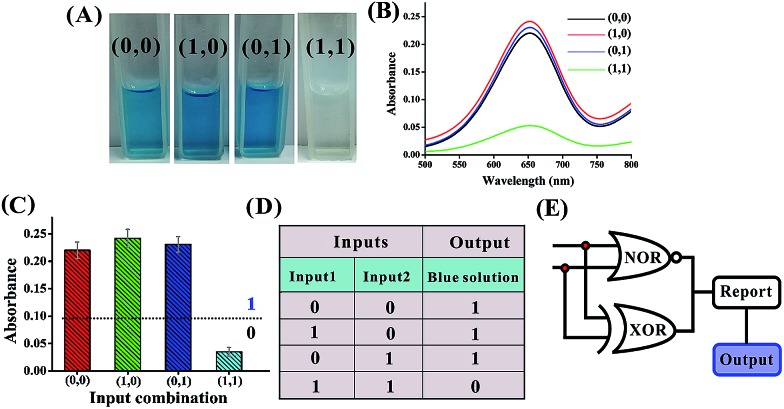
(A) Photographs of the XOR + NOR gate with different combinations of inputs. (B) The absorption spectra of this logic gate. (C) Column diagram of the corresponding absorption intensities at *λ* = 650 nm. The black dashed line shows the threshold value of 0.1. (D) Truth table of the XOR + NOR gate. (E) Electronic equivalent circuitry.

Another integrated circuit composed of parallel XOR and NOR logic gates to enforce an overall NAND logic behavior is also designed. The principle is illustrated in Fig. 9. In the absence of any input, the template DNA1 is hybridized with the DNAzyme subunits (DNA6 and DNA7) to construct an active DNAzyme structure that cleaves the substrate (DNA8) and yields an output of 1. In the presence of either input1 or input2, the input could disassemble the formed active DNAzyme structure from the template DNA1 through the toehold-mediated strand-displacement reaction using segments E and H as the toehold binding domains. However, the subunits (DNA2, DNA3, DNA4 and DNA5) can assemble into another two active DNAzyme structures *via* input-guided cooperative hybridization processes. This leads to the cleavage of the substrate (DNA8) and also yields a true output. In the presence of both inputs, the six DNAzyme subunits (DNA2–DNA7) cannot assemble into any active DNAzyme structure due to the preferred inter-input hybridization and the displacement reactions between the template and the inputs. This results in an output of 0. Thus, parallel activation of a combinatorial circuit established from the XOR and NOR gates could be realized in a single system using the same set of inputs. Fig. 10A shows typical photographs of the XOR + NOR gate. Fig. 10B depicts the absorption spectra from 500 to 800 nm. Fig. 10C shows the corresponding absorption intensity at *λ* = 650 nm. The combinatorial circuit has an output of 0 only if both input signals are 1 and so it behaves like the NAND gate in operation. The truth table and circuitry are given in Fig. 10D and E, respectively.

To demonstrate the feasibility of the logic system for practical applications, the XOR, OR, AND and XOR + AND logic gates were validated in human serum samples. Input1 and input2 in 10% serum were used as the inputs. The results show that the logic system can also execute those computation functions effectively in human serum (Fig. S13–S16, ESI†). These results indicate that the proposed logic system performs well even in relatively complex sample matrices and is not affected when the inputs are in human serum samples.

## Conclusions

In conclusion, a universal set of two-input elementary logic gates (OR, AND, NOR, NAND, INHIBT, IMPLICATION, XOR and XNOR) has been successfully constructed on a label-free and enzyme-free biocomputing platform. Using caged G-quadruplex as the signal transducer, the output of the computation can be unambiguously read out by the naked eye. The uniqueness of the logic system lies in the input-guided assembly and disassembly of the computing circuits, the modularity of the gate design and the visible output which can be obtained by observing the color change of the solution. The study has demonstrated the versatility and scalability of the computing elements by constructing two combinatorial gates. We connect the XOR and AND gates into a multilevel circuit (XOR + AND) that enforces an overall OR gate behavior. Another integrated circuit composed of parallel XOR and NOR logic gates (XOR + NOR) to perform a final net NAND analysis is also designed. The construction strategy described herein is simple in design and economic in operation without any labeling and immobilization procedure or separation and washing step, requiring only the mixing of several solutions at room temperature to obtain the intuitive and visible outputs and so it holds great promise for intelligent POC diagnostics and on-site monitoring.

## Live subject statement

All the experiments involving human serum were approved by the Ethics Committee of Guangdong Institute of Eco-environmental Science & Technology and performed in compliance with the relevant laws and institutional guidelines. We obtained informed consent for any experimentation with human subjects.

## Conflicts of interest

The authors declare no competing financial interest.

## Supplementary Material

Supplementary information
