# Supplementary material for: A label-free and enzyme-free platform with a visible output for constructing versatile logic gates using caged G-quadruplex as the signal transducer
Source: Chem Sci. 2017 Oct 20;9(2):300–6. doi: 10.1039/c7sc04007e (PMC5868315; doi:10.1039/c7sc04007e)
Supplement: Supplementary file 1 [file SC-009-C7SC04007E-s001.pdf]

Electronic Supplementary Material (ESI) for Chemical Science.
This journal is © The Royal Society of Chemistry 2017

Electronic Supplementary Information

Label-free and enzyme-free platform with visible output for constructing versatile logic gates using caged G-quadruplex as the signal transducer

Junhua Chen*, Jiafeng Pan, and Shu Chen

Guangdong Key Laboratory of Integrated Agro-environmental Pollution Control and Management, Guangdong Institute of Eco-environmental Science & Technology, Guangzhou 510650, China

*Corresponding author:

E-mail: 222chenjunhua@163.com

Experimental Section

Chemicals and materials

Hemin, 3,3',5,5'-tetramethylbenzidine (TMB), hydrogen peroxide (H_2O_2, 30wt.%), Triton X-100, dimethyl sulfoxide (DMSO), and tris-(hydroxymethyl)aminomethane (Tris) were purchased from Sigma-Aldrich (St. Louis, MO). A hemin stock solution (5 mM) was prepared in DMSO and stored in the dark at -20 ^{o}C. Other reagents and chemicals were of analytical grade and used without purification. All solution was prepared with ultrapure water (18.2 MΩ/cm) from a Millipore Milli-Q water purification system (Billerica, MA).

All DNA sequences were designed to minimize undesired cross-hybridization using NUPACK (http://www. nupack.org/).[1,2] DNA oligonucleotides were HPLC-purified and purchased from Shanghai Sangon Biotechnology Co., Ltd. (Shanghai, China) and their sequences were listed as follows:

DNA sequences for AND gate:

C A

Input1: 5'-TATGACACATCAC-CTACAAATACCTA-3'

B C'

Input2: 5'-CGAATCCTGAGT-GTGATGTGTCATA-3'

A' I M

DNA1: 5'-TAGGTATTTGTAG-GTTACACCCATGT-CATTCA-3'

N II B'

DNA2: 5'-GATATC-AGCGATTAAC-ACTCAGGATTCG-3'

M' N'

DNA3: 5'-CCTACCC-TGAATGTrAGGATATC-TATTTTTTTTTATGGGTAGGGCGGGTTGGG-3'

DNA sequences for OR gate:

B A

Input1: 5'-CGAATCCTGAGT-CTACAAATACCTA-3'

D C

Input2: 5'-GCAGACTACTACT-TATGACACATCAC-3'

A' I M

DNA1: 5'-TAGGTATTTGTAG-GTTACACCCATGT-CATTCA-3'

N II B'

DNA2: 5'-GATATC-AGCGATTAAC-ACTCAGGATTCG-3'

C' I M

DNA3: 5'-GTGATGTGTCATA-GTTACACCCATGT-CATTCA -3'

N II D'
DNA4: 5'-GATATC-AGCGATTAAC-AGTAGTAGTCTGC-3'

M' N'
DNA5: 5'-CCTACCC-TGAATGTrAGGATATC-TATTTTTTTTTATGGGTAGGGCGGGTTGGG-3'

DNA sequences for INHIBIT gate:

B A
Input1: 5'-CGAATCCTGAGT-CTACAAATACCTA-3'

A' B'
Input2: 5'- TAGGTATTTGTAG-ACTCAGGATTCG-3'

A' I M
DNA1: 5'-TAGGTATTTGTAG-GTTACACCCATGT-CATTCA-3'

N II B'
DNA2: 5'-GATATC-AGCGATTAAC-ACTCAGGATTCG-3'

M' N'
DNA3: 5'-CCTACCC-TGAATGTrAGGATATC-TATTTTTTTTTATGGGTAGGGCGGGTTGGG-3'

DNA sequences for IMPLICATION gate:

C' A'
Input1: 5'-GTGTCATA- TAGGTATTTGTAG-3'

A C
Input2: 5'-CTACAAATACCTA-TATGACAC-3'

B A C
DNA1: 5'-CGAATCCTGAGT-CTACAAATACCTA-TATGACAC 3'

A' I M
DNA2: 5'-TAGGTATTTGTAG-GTTACACCCATGT-CATTCA-3'

N II B'
DNA3: 5'-GATATC-AGCGATTAAC-ACTCAGGATTCG-3'

M' N'
DNA4: 5'-CCTACCC-TGAATGTrAGGATATC-TATTTTTTTTTATGGGTAGGGCGGGTTGGG-3'

DNA sequences for NOR gate:

C' A'
Input1: 5'-GTGTCATA-TAGGTATTTGTAG-3'

B' D'
Input2: 5'-ACTCAGGATTCG-AGTAGTAG-3'

D B A C
DNA1: 5'-CTACTACT-CGAATCCTGAGT-CTACAAATACCTA-TATGACAC-3'

A' I M
DNA2: 5'-TAGGTATTTGTAG-GTTACACCCATGT-CATTCA-3'

N II B'

DNA3: 5'-GATATC-AGCGATTAAC-ACTCAGGATTCG-3'

M' N'

DNA4: 5'-CCTACCC-TGAATGTrAGGATATC-TATTTTTTTTTATGGGTAGGGCGGGTTGGG-3'

DNA sequences for NAND gate:

C' A'

Input1: 5'-GTGTCATA-TAGGTATTTGTAG-3'

E' F'

Input1: 5'-GACTCGTATGC-AACTGGTG-3'

F E D B

DNA1: 5'-CACCAGTT-GCATACGAGTC-GCAGACTACTACT-CGAATCCTGAGT-

A C

CTACAAATACCTA-TATGACAC-3'

A' I M

DNA2: 5'-TAGGTATTTGTAG-GTTACACCCATGT-CATTCA-3'

N II B'

DNA3: 5'-GATATC-AGCGATTAAC-ACTCAGGATTCG-3'

D' I M

DNA4: 5'-AGTAGTAGTCTGC-GTTACACCCATGT-CATTCA-3'

N II E'

DNA5: 5'-GATATC-AGCGATTAAC-GACTCGTATGC-3'

M' N'

DNA6: 5'-CCTACCC-TGAATGTrAGGATATC-TATTTTTTTTTATGGGTAGGGCGGGTTGGG-3'

DNA sequences for XOR gate:

D C B A

Input1: 5'-GCAGACTACTACT-TATGACACATCAC-CGAATCCTGAGT-CTACAAATACCTA-3'

A' B' C' D'

Input2: 5'-TAGGTATTTGTAG-ACTCAGGATTCG-GTGATGTGTCATA-AGTAGTAGTCTGC-3'

A' I M

DNA1: 5'-TAGGTATTTGTAG-GTTACACCCATGT-CATTCA-3'

N II B'

DNA2: 5'-GATATC-AGCGATTAAC-ACTCAGGATTCG-3'

D I M

DNA3: 5'- GCAGACTACTACT-GTTACACCCATGT-CATTCA -3'

N II C

DNA4: 5'-GATATC-AGCGATTAAC-TATGACACATCAC-3'

M' N'

DNA5: 5'-CCTACCC-TGAATGTrAGGATATC-TATTTTTTTTTATGGGTAGGGCGGGTTGGG-3'

DNA sequences for XNOR gate:

C' A' E F
Input1: 5'-GTGTCATA-TAGGTATTTGTAG-GCATACGAGTC-TAGCACCAGTT-3'

G E' B' D'
Input2: 5'-CAAGGCAGGATT-GACTCGTATGC-ACTCAGGATTCG-AGTAGTAG-3'

D B A C
DNA1: 5'-CTACTACT-CGAATCCTGAGT-CTACAAATACCTA-TATGACAC-3'

A' I M
DNA2: 5'-TAGGTATTTGTAG-GTTACACCCATGT-CATTCA-3'

N II B'
DNA3: 5'-GATATC-AGCGATTAAC-ACTCAGGATTCG-3'

F' I M
DNA4: 5'-AACTGGTGCTA-GTTACACCCATGT-CATTCA-3'

N II G'
DNA5: 5'-GATATC-AGCGATTAAC-AATCCTGCCTTG-3'

M' N'
DNA6: 5'-CCTACCC-TGAATGTrAGGATATC-TATTTTTTTTTATGGGTAGGGCGGGTTGGG-3'

DNA sequences for XOR+AND gate:

E D C B
Input1: 5'-GCATACGAGTC-GCAGACTACTACT-TATGACACATCAC-CGAATCCTGAGT-
A
CTACAAATACCTA-3'

A' B' C' D'
Input2: 5'-TAGGTATTTGTAG-ACTCAGGATTCG-GTGATGTGTCATA-AGTAGTAGTCTGC-
F
TAGCACCAGTT-3'

A' I M
DNA1: 5'-TAGGTATTTGTAG-GTTACACCCATGT-CATTCA-3'

N II B'
DNA2: 5'-GATATC-AGCGATTAAC-ACTCAGGATTCG-3'

D I M
DNA3: 5'- GCAGACTACTACT-GTTACACCCATGT-CATTCA -3'

N II C
DNA4: 5'-GATATC-AGCGATTAAC-TATGACACATCAC-3'

F' I M
DNA5: 5'-AACTGGTGCTA-GTTACACCCATGT-CATTCA-3'

N II E'
DNA6: 5'-GATATC-AGCGATTAAC-GACTCGTATGC-3'

M' N'
DNA7: 5'-CCTACCC-TGAATGTrAGGATATC-TATTTTTTTTTATGGGTAGGGCGGGTTGGG-3'

DNA sequences for XOR+NOR gate:

F' E' D C

Input1: 5'-AACTGGTGCTA-TCGTATGC-GCAGACTACTACT-TATGACACATCAC-

B A

CGAATCCTGAGT-CTACAAATACCTA-3'

A' B' C' D'

Input2: 5'-TAGGTATTTGTAG-ACTCAGGATTCG-GTGATGTGTCATA-AGTAGTAGTCTGC-3'

G' H'

AATCCTGCCTTG-AGATACAT-3'

H G F E

DNA1: 5'-ATGTATCT-CAAGGCAGGATT-TAGCACCAGTT-GCATACGA-3'

A' I M

DNA2: 5'-TAGGTATTTGTAG-GTTACACCCATGT-CATTCA-3'

N II B'

DNA3: 5'-GATATC-AGCGATTAAC-ACTCAGGATTCG-3'

D I M

DNA4: 5'- GCAGACTACTACT-GTTACACCCATGT-CATTCA -3'

N II C

DNA5: GATATC-AGCGATTAAC-TATGACACATCAC-3'

F' I M

DNA6: 5'-AACTGGTGCTA-GTTACACCCATGT-CATTCA-3'

N II G'

DNA7: GATATC-AGCGATTAAC-AATCCTGCCTTG-3'

M' N'

DNA8: 5'-CCTACCC-TGAATGTrAGGATATC-TATTTTTTTTTATGGGTAGGGCGGGTTGGG-3'

General procedures for logic gate operation

All assays were performed in 20 mM Tris-HCl buffer (pH = 7.4, 200 mM NaCl, 20 mM $MgCl_2$) at a final DNA concentration of 1 μM. The samples, without the inputs, were heated to 90 °C for 10 min, and then cooled down to room temperature for 90 min. The input was added to the above solution, and the mixture was incubated for 120 min at room temperature. The resulting solution was used for subsequent colorimetric detection.

Colorimetric analysis

10 μL of 20 mM Tris-HCl buffer solution (pH = 7.4, containing 2 μM hemin, 15 mM KCl, 5 mM $MgCl_2$, 0.03% Triton X-100, and 1% DMSO) was added to 40 μL of the above solution, and the mixture was incubated at room temperature for 45 min to form the active hemin/G-quadruplex DNAzymes. In a typical catalytic reaction, 50 μL of the final DNA-hemin mixture solution was mixed with 950 μL of TMB-H_2O_2 substrate solution, which was constituted of 10 μL 0.5% (w/v) TMB, 20 μL 30% (w/v) H_2O_2, and 920 μL substrate buffer (containing 26.6 mM citric acid, 51.4 mM disodium hydrogen phosphate, and 15 mM KCl, pH = 5.0). After incubation at room temperature for 15 min, the colorimetric responses were observed by the naked eye and the photos were taken by a digital camera. Meanwhile, the absorption intensity of the solution were recorded by a TU-1810 UV-vis spectrophotometer.

Circular dichroism (CD) experiment

CD spectra were measured on a Chirascan Circular Dichroism Spectrometer (Applied Photophysics Ltd, England, UK) at room temperature. The DNA solution (3 μM) was prepared in 20 mM Tris-HAc buffer (pH 7.4) containing 50 mM KAc and 50 mM $Mg(NO_3)_2$. The solution was heated to 90 °C for 5 min, gradually cooled to room, and incubated at RT for 3h. After incubation with 3 μM hemin for 1 h, CD spectra from 320 to 220 nm were recorded in 1 mm path length cuvettes and averaged from three scans with the buffer background subtracted.

Effects of pH and ion strength

To investigate the effects of pH and ion strength on the response of the logic system, the (1,1) state of the AND logic gate was tested under different pH and ion strength. As shown in Fig. S1, a wide range of pH (from 5 to 9) and ion strength (with NaCl concentrations from 50 to 400 mM) did not change the logic functions of the computation states, indicating the robustness of this logic system which is beneficial for practical applications.

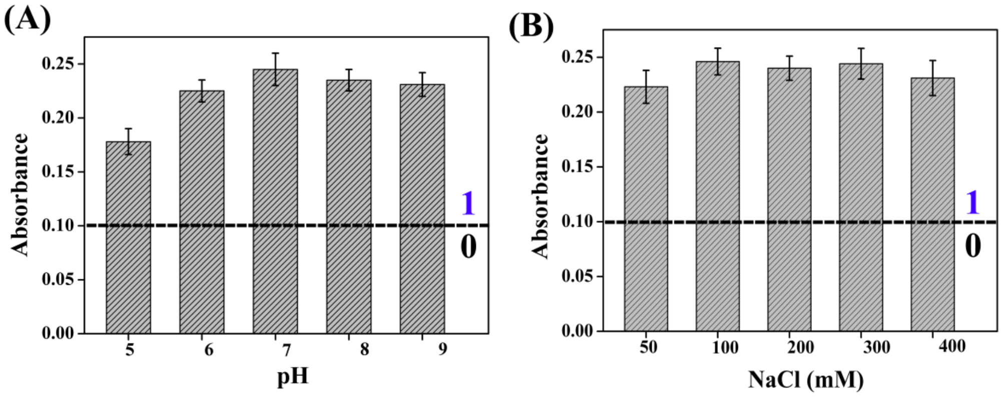

Fig. S1 Effects of pH (A) and ionic strength (B) on the response of the logic system, with the pH values varying from 5 to 9 and the concentrations of NaCl changing from 50 to 400 mM. The error bars represent the standard deviation of three independent measurements. The black dashed line shows the threshold value of 0.1.

Circular dichroism results

Taking the AND logic gate as an example, the formation of the G-quadruplex structure was verified by circular dichroism (CD) spectroscopy. As shown in Fig. S2, there was no obvious peak in the (0,0) state of the AND gate. In the (1,0) or (0,1) state, the intermediate DNA structure showed a positive peak near 275 nm and a negative peak near 245 nm, which referred to the typical characteristic of double helix structure.[3,4] In the presence of both inputs (1,1), the final DNA structure had two characteristic peaks at 264 and 240 nm. The positions of the positive and negative peaks match the reported characteristic peaks of the parallel G-quadruplex,[5,6] indicating the successful formation of the G-quadruplex structure in the (1,1) state of the AND logic gate.

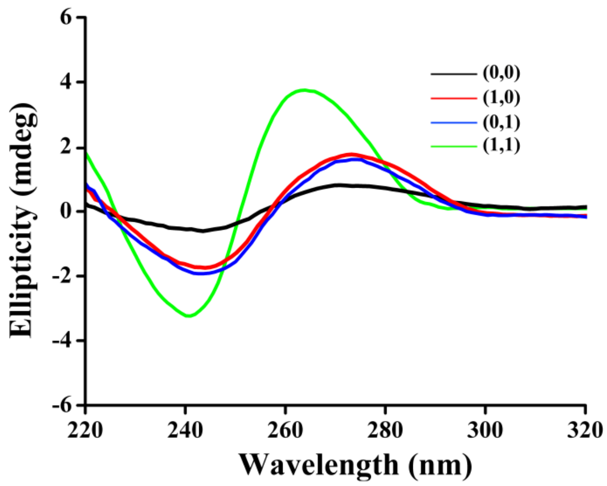

Fig. S2 CD spectra for analyzing the DNA structure in the AND logic gate under different input combinations. Concentrations of the DNA strands were 3 μM.

XNOR logic gate

The construction of the XNOR logic gate is outlined in Fig. S3. An additional DNA template (DNA1) is introduced to bind with the DNAzyme subunits (DNA2 and DNA3) to form an active DNAzyme. This original state (0,0) results in the cleavage of the substrate (DNA6), releases the caged G-quadruplex sequence, and gives rise to the output of 1. The addition of either input could disassemble the active DNAzyme structure and the output is 0. The overhung segments C and D in DNA1 serve as the toehold domains and proceed to invade and displace the previously hybridized DNAzyme subunits *vis* the toehold-mediated strand-displacement reaction in presence of input1 (1,0) or input2 (0,1). If both inputs are present (1,1), the partial duplex between input1 and input2 is formed (domain E is complementary with domain E'). The cooperative interactions of the DNAzyme subunits (DNA4 and DNA5) with the input1-input2 yields the assembly of another active DANzyme, consequently leading to the cleavage of DNA6 and resulting in blue solution. Fig. S4A presents typical photo images of the XNOR gate. Fig. S4B depicts absorption spectra from 500 to 800 nm. Fig. S4C shows the corresponding absorption intensity at λ = 650 nm. This gate has an output of 1 (blue solution) only if the two inputs are present or absent. The truth table and circuitry are given in Fig. S4D and S4E, respectively.

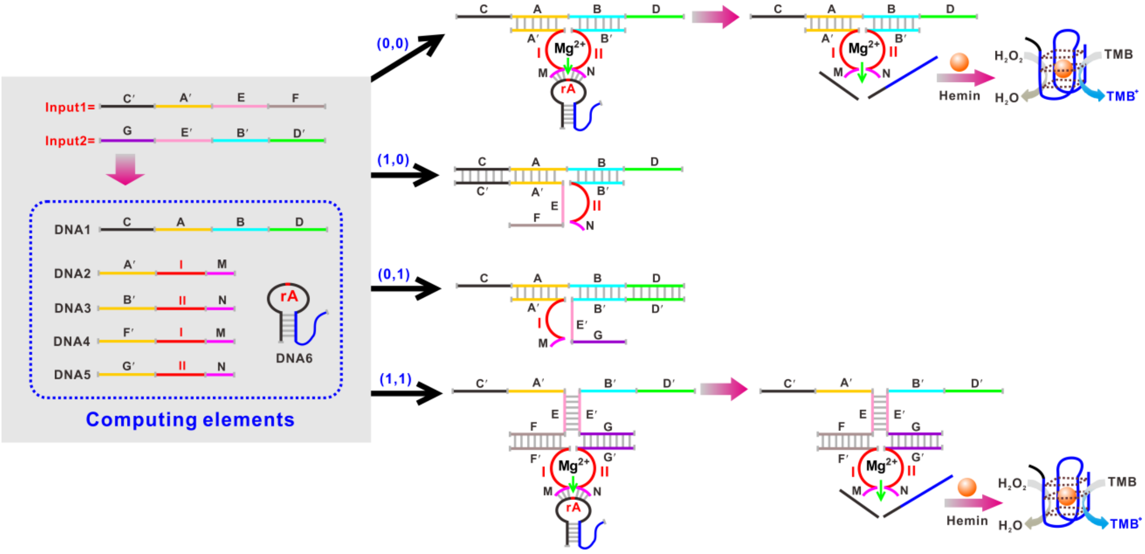

Fig. S3 Schematic representation of the XNOR logic gate that consists of the DNA template (DNA1), the DNAzyme subunits (DNA2-DNA5), the substrate (hairpin DNA6, the caged G-quadruplex sequence in the stem structure of the hairpin is indicated in blue), and the input DNA.

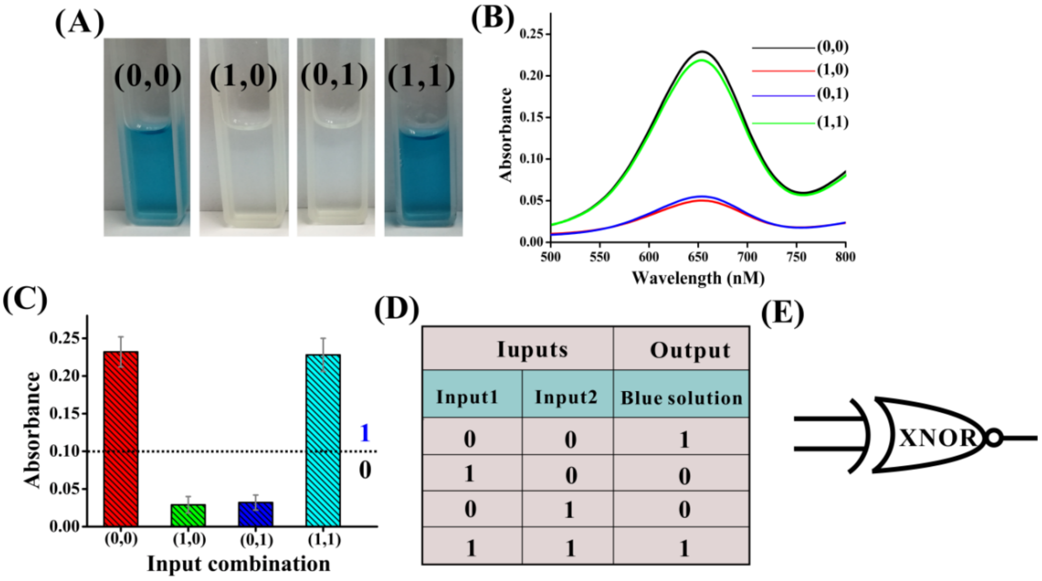

Iuputs		Output
Input1	Input2	Blue solution
0	0	1
1	0	0
0	1	0
1	1	1

Fig. S4 (A) Photographs of the XNOR gate with different combinations of inputs. (B) The absorption spectra of this logic gate. (C) Column diagram of the corresponding absorption intensities at $\lambda = 650$ nm. The black dashed line shows the threshold value of 0.1. (D) Truth table of the XNOR gate. (E) Electronic equivalent circuitry.

NAND logic gate

A NAND gate is the inverted result of an AND gate, which causes all 0 output to switch to 1 and vice versa. As shown in Fig. S5, in the absence of any input, the template DNA1 bridges the DNAzyme subunits (DNA2-DNA4) to assemble two different active DNAzymes, leading to the cleavage of the mutual substrate DNA6 and to the generation of a true output. In the presence of either input1 or input2, the input-initiated toehold-mediated strand-displacement reaction could disassemble one of the formed two active DNAzyme structure using segments C and F as the toehold binding domains. The other DNAzyme keeps intact and active that leads to the cleavage of the substrate and also yields a true output. In the presence of both inputs, the hybridization of the inputs to the template DNA1 can eliminate the two active DNAzymes, leading to a false output. Fig. S6A presents typical photo images of the NAND gate. Fig. S6B depicts absorption spectra from 500 to 800 nm. Fig. S6C shows the corresponding absorption intensity at λ = 650 nm. The NAND logic gate has an output of 0 only if both inputs are 1. The truth table and circuitry are given in Fig. S6D and S6E, respectively.

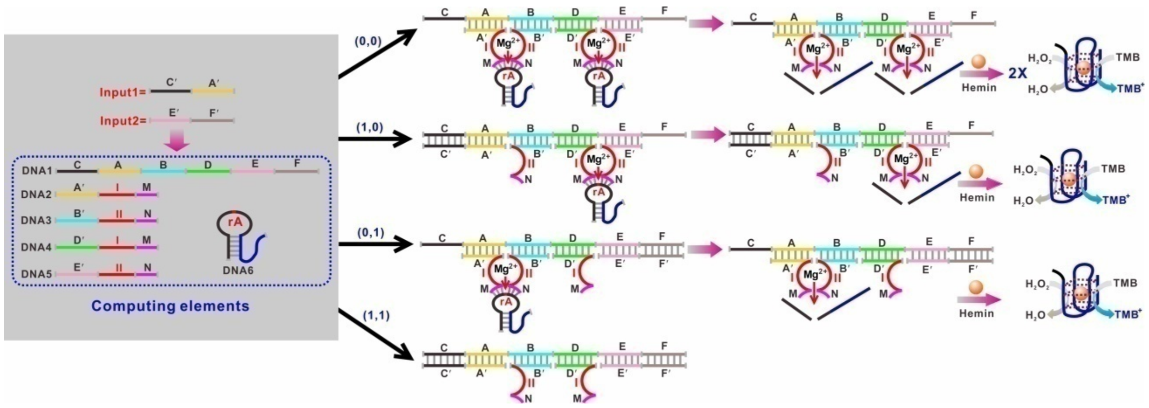

Fig. S5 Schematic representation of the NAND logic gate that consists of the DNA template (DNA1), the DNAzyme subunits (DNA2-DNA5), the substrate (hairpin DNA6, the caged G-quadruplex sequence in the stem structure of the hairpin is indicated in blue), and the input DNA.

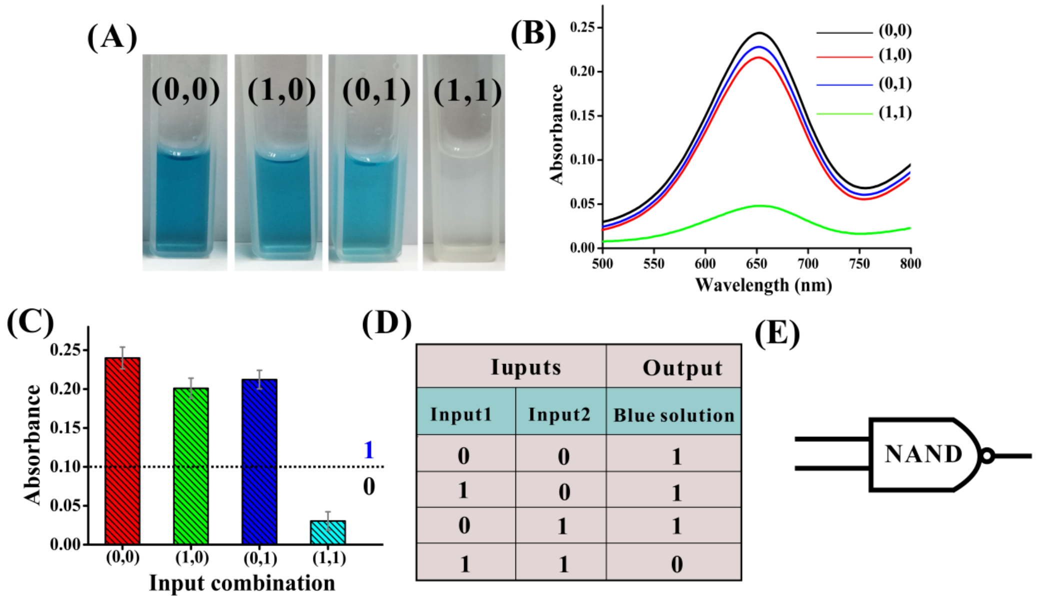

Iuputs		Output
Input1	Input2	Blue solution
0	0	1
1	0	1
0	1	1
1	1	0

Fig. S6 (A) Photographs of the NAND gate with different combinations of inputs. (B) The absorption spectra of this logic gate. (C) Column diagram of the corresponding absorption intensities at λ = 650 nm. The black dashed line shows the threshold value of 0.1. (D) Truth table of the NAND gate. (E) Electronic equivalent circuitry.

NOR logic gate

A NOR gate is the result of the negation of an OR gate. As illustrated in Fig. S7, in the (0,0) state, the template DNA1 is hybridized with the DNAzyme subunits (DNA2 and DNA3) to construct the active DNAzyme structure that cleaves the substrate (DNA4) and yields an output of 1. In the presence of either input1 or input2, the input could disassemble the active DNAzyme structure from the template *vis* the toehold-mediated strand-displacement reaction using segments C and D as the toehold binding domains. This leads to the false output of the gate. Similarly, in the presence of both inputs, the template is more favorably hybridized with the inputs to form duplexes by displacement mechanism. This process results in the dissociation of the active DNAzyme, consequently leading to an output of 0. Fig. S8A presents typical photo images of the NOR gate. Fig. S8B depicts absorption spectra from 500 to 800 nm. Fig. S8C shows the corresponding absorption intensity at λ = 650 nm. The NOR logic gate has an output of 1 only if both inputs are 0. The truth table and circuitry are given in Fig. S8D and S8E, respectively.

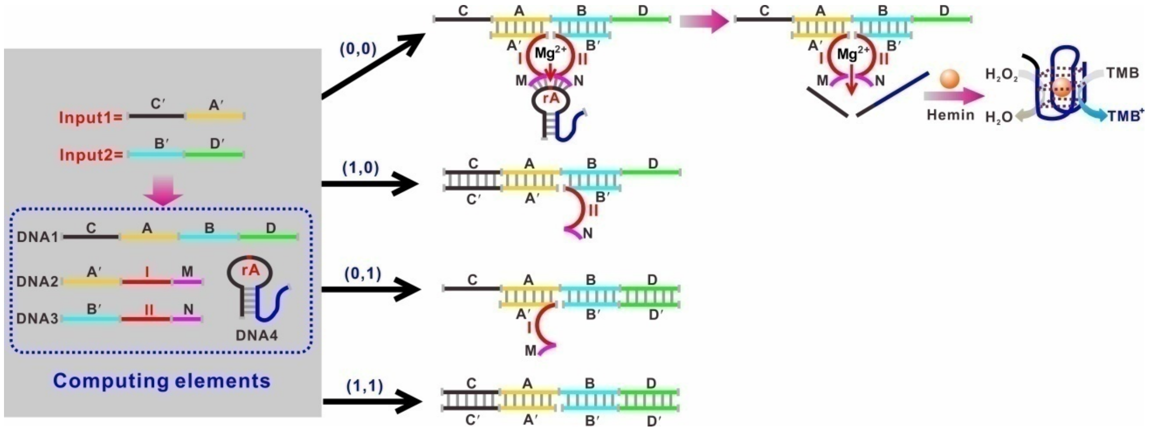

Fig. S7 Schematic representation of the NOR logic gate that consists of the DNA template (DNA1), the DNAzyme subunits (DNA2 and DNA3), the substrate (hairpin DNA4, the caged G-quadruplex sequence in the stem structure of the hairpin is indicated in blue), and the input DNA.

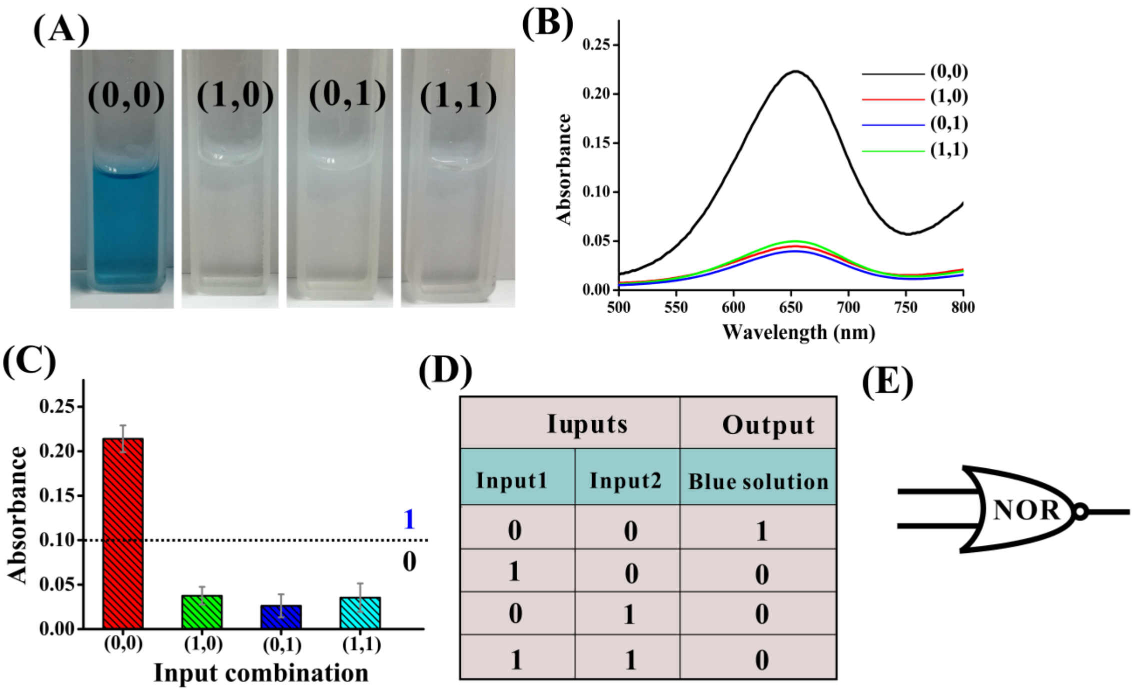

Fig. S8 (A) Photographs of the NOR gate with different combinations of inputs. (B) The absorption spectra of this logic gate. (C) Column diagram of the corresponding absorption intensities at λ = 650 nm. The black dashed line shows the threshold value of 0.1. (D) Truth table of the NOR gate. (E) Electronic equivalent circuitry.

INHIBIT logic gate

An INHIBIT logic gate, which consists of two DNAzyme subunits (DNA1 and DNA2), the substrate DNA3, and the input DNA, is also designed. As illustrated in Fig. S9, in the presence of input1 (1,0), an active DANzyme is formed, leading to the cleavage of the substrate and to the generation of a blue color signal. In the presence of another input (0,1), the system fails to yield any active DANzyme for readout because of the identical DNA sequences between the recognition arms (segments A' and B') of the DNAzyme subunits and the input2. Triggering the system with both inputs (1,1) results in the formation of the duplex structure between input1 and input2. This prohibits the formation of an active DANzyme and the generation of a true output. Fig. S10A presents typical photo images of the INHIBIT gate. Fig. S10B depicts absorption spectra from 500 to 800 nm. and Fig. S10C shows the corresponding absorption intensity at λ = 650 nm. These results are consistent with the execution of an INHIBIT gate which is represented by the situation where the output is 1 only if one input is 1; otherwise, the ouput is 0. The truth table and circuitry are given in Fig. S10D and S10E, respectively.

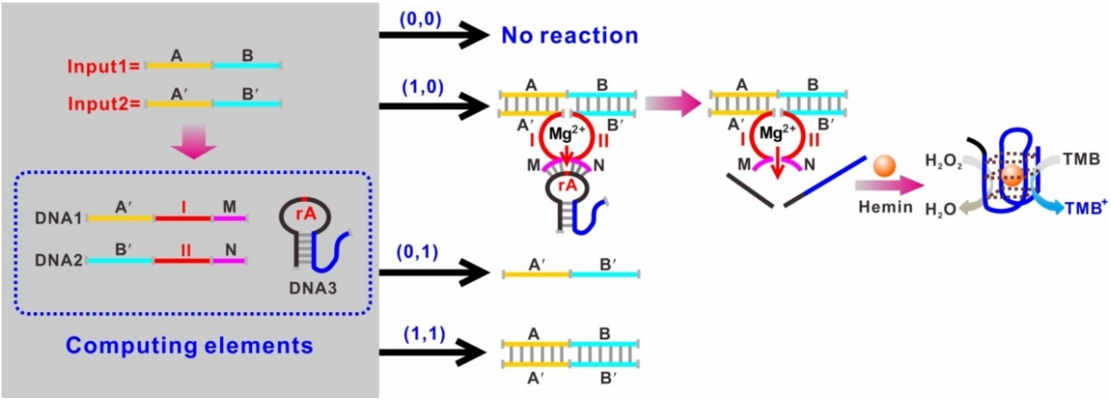

Fig. S9 Schematic representation of the INHIBIT logic gate that consists of the DNAzyme subunits (DNA1 and DNA2), the substrate (hairpin DNA3, the caged G-quadruplex sequence in the stem structure of the hairpin is indicated in blue), and the input DNA.

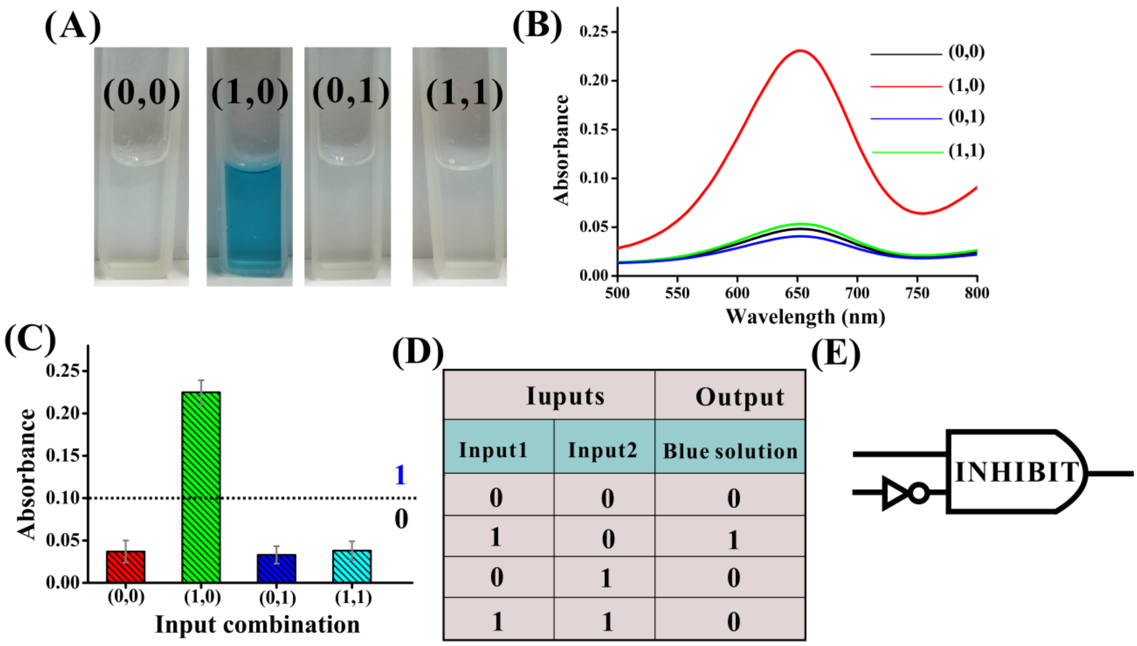

Fig. S10 (A) Photographs of the INHIBIT gate with different combinations of inputs. (B) The absorption spectra of this logic gate. (C) Column diagram of the corresponding absorption intensities at λ = 650 nm. The black dashed line shows the threshold value of 0.1. (D) Truth table of the INHIBIT gate. (E) Electronic equivalent circuitry.

IMPLICATION logic gate

An IMPLICATION logic gate is the reverse output result of an INHIBIT logic gate. As shown in Fig. S11, an additional DNA template (DNA1) can hybridize with the DNAzyme subunits (DNA2 and DNA3) to form an active DNAzyme, giving rise to the output of 1 in the absence of input. To trigger the disassembly of the active DNAzyme, the input1 (1,0) is introduced to initiate the toehold-mediated strand-displacement reaction to displace one DNAzyme subunit (DNA2) from the DNA template. As expected, the disassembly of the active DNAzyme results in a false output. In the (0,1) and (1,1) combinations, the strand displacement reactions are prohibited for the disassembly processes, because there is no toehold binding between the input and the template DNA. Thus, the formed active DNAzyme keeps the intact structure for the cleavage of the substrate. Fig. S12A presents typical photo images of the IMPLICATION gate. Fig. S12B depicts absorption spectra from

500 to 800 nm. Fig. S12C shows the corresponding absorption intensity at λ = 650 nm. The IMPLICATION logic gate provides the true output of 1 in all circumstances, except for the situation that one particular input is 1. The truth table and circuitry are given in Fig. S12D and S12E, respectively.

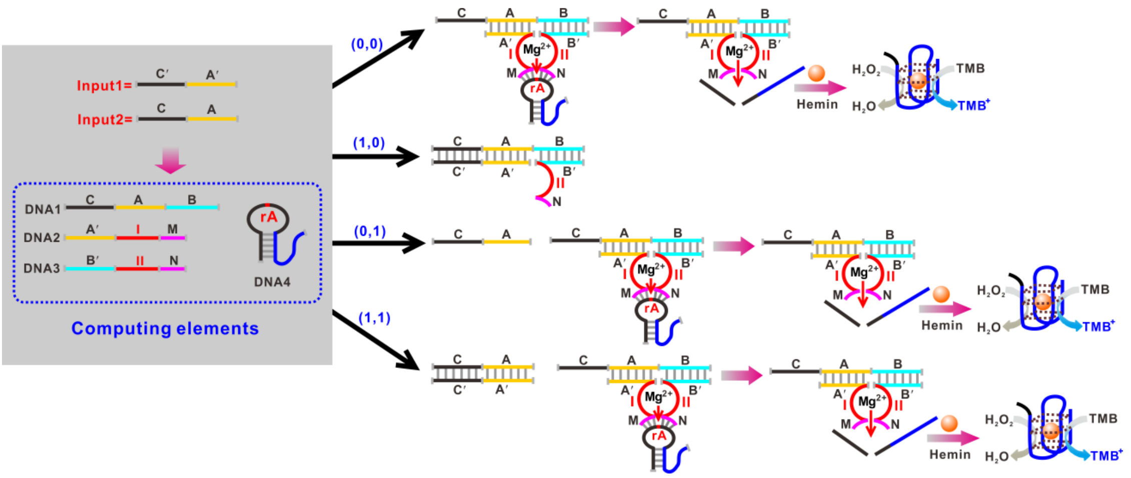

Fig. S11 Schematic representation of the IMPLICATION logic gate that consists of the DNA template (DNA1), the DNAzyme subunits (DNA2 and DNA3), the substrate (hairpin DNA4, the caged G-quadruplex sequence in the stem structure of the hairpin is indicated in blue), and the input DNA.

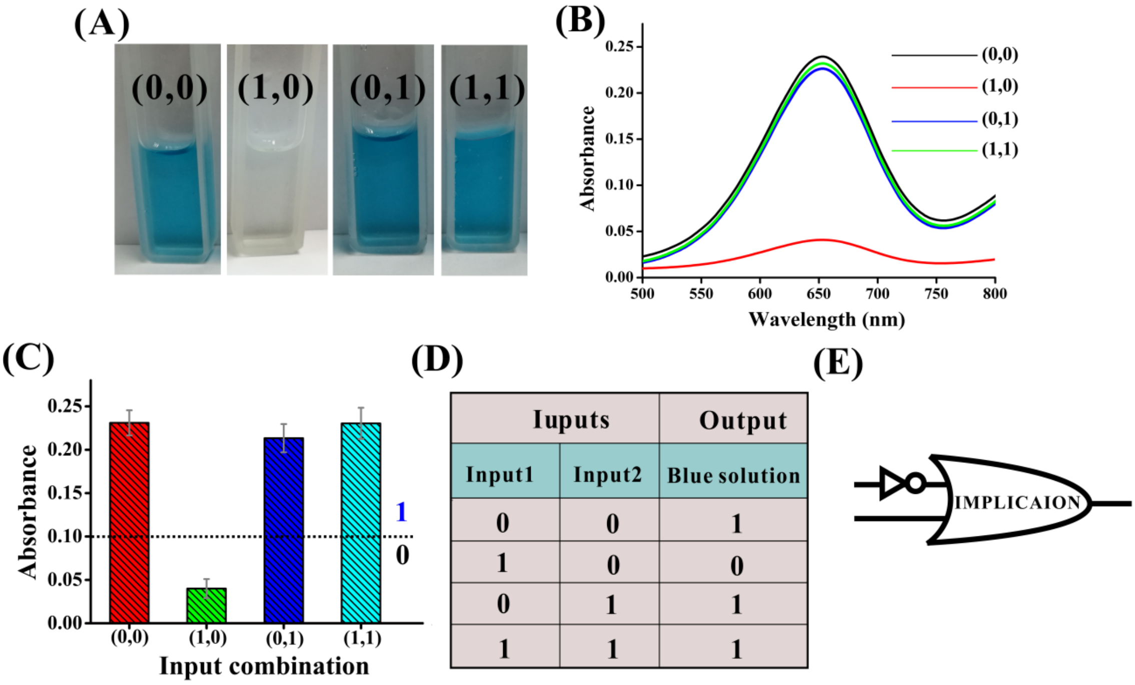

Iuputs		Output
Input1	Input2	Blue solution
0	0	1
1	0	0
0	1	1
1	1	1

Fig. S12 (A) Photographs of the IMPLICATION gate with different combinations of inputs. (B) The absorption spectra of this logic gate. (C) Column diagram of the corresponding absorption intensities at λ = 650 nm. The black dashed line shows the threshold value of 0.1. (D) Truth table of the IMPLICATION gate. (E) Electronic equivalent circuitry.

Practical applications of the logic gates

To demonstrate the feasibility of the logic system for practical applications, the XOR, OR, AND, and XOR+AND logic gates were validated in human serum samples. Serum samples were prepared by diluting to 10% in dilution buffer (20 mM Tris-HCl, 200 mM NaCl, 20 mM $MgCl_2$, pH 7.4). The input1 and input2 spiked in 10% human serum were used as inputs to perform the logic operations. As shown in Fig. S13A, in the presence of either spiked input1 (1,0) or input2 (0,1), the solution turned blue (output = 1). In the absence of input (0,0) or in the presence of both spiked inputs (1,1), the logic gate failed to generate a blue solution (output = 0). Those behaviors corresponded to the XOR logic gate. Fig. S13B depicts absorption spectra from 500 to 800 nm. Fig. S13C shows the corresponding absorption intensity at λ = 650 nm. The truth table and circuitry are given in Fig. S13D and S13E, respectively. As shown in Fig. S14A, without the spiked input1 and input2 (0,0), the solution is colorless in the OR gate, but when serum samples were spiked with either input1 (1,0) or input2 (0,1) or both inputs (1,1), the blue color solution can be observed. Fig. S14B depicts absorption spectra from 500 to 800 nm. Fig. S14C shows the corresponding absorption intensity at λ = 650 nm. The truth table and circuitry are given in Fig. S14D and S14E, respectively. As shown in Fig. S15A, the blue color solution was generated only when the AND gate was subjected to the two spiked inputs together (1,1). The other three input combinations all fail to produce an obvious colorimetric response. Fig. S15B depicts absorption spectra from 500 to 800 nm. Fig. S15C shows the corresponding absorption intensity at λ = 650 nm. The truth table and circuitry are given in Fig. S15D and S15E, respectively. As shown in Fig. S16A, in the presence of either spiked input1 (1,0) or input2 (0,1), the solution turned blue (output = 1), which corresponded to the XOR logic gate. When the integrated circuit was subjected to the two spiked inputs together (1,1), the solution also turned blue, which corresponded to the AND logic gate. Thus, an integrated circuit established form the XOR and AND gates could be performed parallelly in human serum samples. Fig. S16B depicts absorption spectra from 500 to 800 nm. Fig. S16C shows the corresponding absorption intensity at λ = 650 nm. The truth table and circuitry are given in Fig. S16D and S16E, respectively. These results indicate that the proposed logic system performs well even in relatively complex sample matrixes and is not affected when the inputs existed in human serum samples, which holds great promise for POC diagnostics and in-field applications.

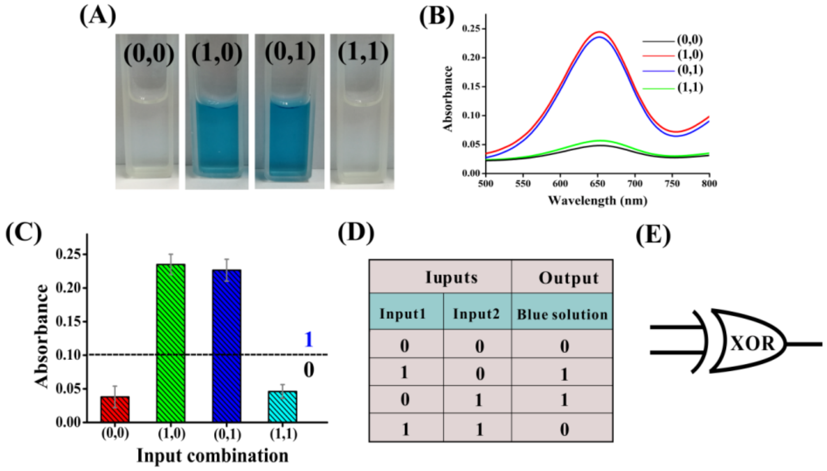

Fig. S13 The results of the application of the XOR logic gate in 10% human serum samples. (A) Photographs of the XOR gate with different combinations of inputs. (B) The absorption spectra of this logic gate. (C) Column diagram of the corresponding absorption intensities at λ = 650 nm. The black dashed line shows the threshold value of 0.1. (D) Truth table of the XOR gate. (E) Electronic equivalent circuitry.

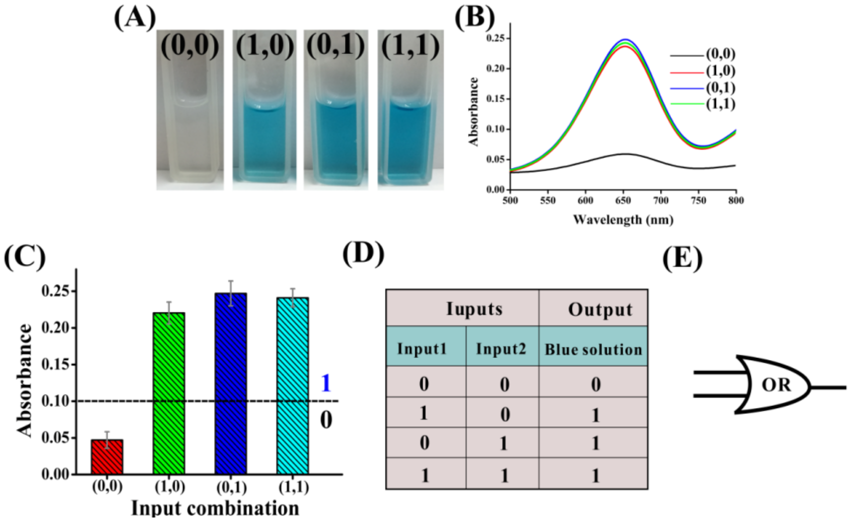

Fig. S14 The results of the application of the OR logic gate in 10% human serum samples. (A) Photographs of the OR gate with different combinations of inputs. (B) The absorption spectra of this logic gate. (C) Column diagram of the corresponding absorption intensities at λ = 650 nm. The black dashed line shows the threshold value of 0.1. (D) Truth table of the OR gate. (E) Electronic equivalent circuitry.

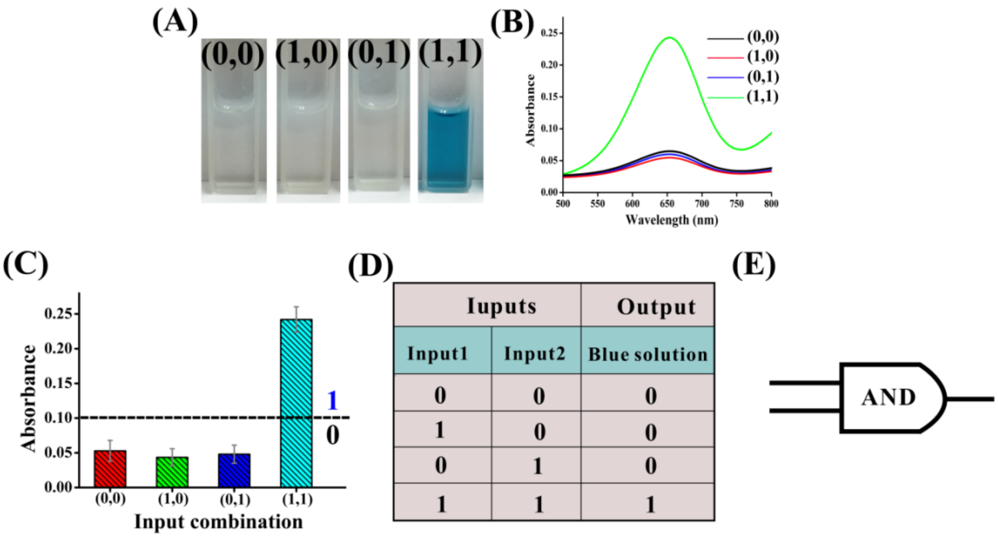

Iuputs		Output
Input1	Input2	Blue solution
0	0	0
1	0	0
0	1	0
1	1	1

Fig. S15 The results of the application of the AND logic gate in 10% human serum samples. (A) Photographs of the AND gate with different combinations of inputs. (B) The absorption spectra of this logic gate. (C) Column diagram of the corresponding absorption intensities at λ = 650 nm. The black dashed line shows the threshold value of 0.1. (D) Truth table of the AND gate. (E) Electronic equivalent circuitry.

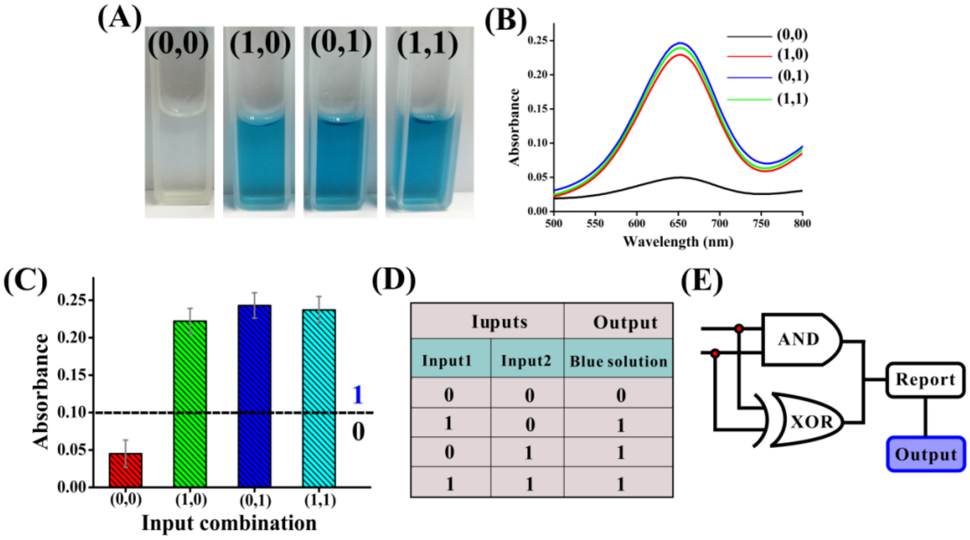

Iuputs		Output
Input1	Input2	Blue solution
0	0	0
1	0	1
0	1	1
1	1	1

Fig. S16 The results of the application of the XOR+AND logic gate in 10% human serum samples. (A) Photographs of the XOR+AND gate with different combinations of inputs. (B) The absorption spectra of this logic gate. (C) Column diagram of the corresponding absorption intensities at λ = 650 nm. The black dashed line shows the threshold value of 0.1. (D) Truth table of the XOR+AND gate. (E) Electronic equivalent circuitry.

References

[1] R. M. Dirks, J. S. Bois, J. M. Schaeffer, E. Winfree and N. A. Pierce, *SIAM Rev.,* 2007, **49**, 65-88.

[2] J. N. Zadeh, B. R. Wolfe and N. A. Pierce, *J. Comput. Chem.,* 2011, **32**, 439-452.

[3] J. Zhu, T. Li, L. Zhang, S. Dong and E. Wang, *Biomaterials* 2011, **32**, 7318-7324.

[4] Y. Guo, J. Wu and H. Ju, *Chem. Sci.,* 2015, **6** , 4318-4323.

[5] Y. Zhao, Q. Zhang, W. Wang and Y. Jin, *Biosens. Bioelectron.,* 2013, **43**, 231-236.

[6] J. Zhu, L. Zhang, S. Dong and E. Wang, *Chem. Sci.,* 2015, **6** , 4822-4827.
